# Linking behavioral states to landscape features for improved conservation management

**DOI:** 10.1002/ece3.7621

**Published:** 2021-05-25

**Authors:** Maitreyi Sur, Brian Woodbridge, Todd C. Esque, Jim R. Belthoff, Peter H. Bloom, Robert N. Fisher, Kathleen Longshore, Kenneth E. Nussear, Jeff A. Tracey, Melissa A. Braham, Todd E. Katzner

**Affiliations:** ^1^ Conservation Science Global, Inc. West Cape May NJ USA; ^2^ Boise State University Boise ID USA; ^3^ U.S. Fish and Wildlife Service Corvallis OR USA; ^4^ U.S. Geological Survey Western Ecological Research Center Henderson NV USA; ^5^ Bloom Research Inc. Los Angeles CA USA; ^6^ U.S. Geological Survey Western Ecological Research Center San Diego CA USA; ^7^ Department of Geography University of Nevada – Reno Reno NV USA; ^8^ U.S. Geological Survey Forest and Rangeland Ecosystem Science Center Boise ID USA

**Keywords:** animal movement, behavioral change point analysis, conservation management, Golden Eagle, GPS telemetry

## Abstract

1. A central theme for conservation is understanding how animals differentially use, and are affected by change in, the landscapes they inhabit. However, it has been challenging to develop conservation schemes for habitat‐specific behaviors.
2. Here we use behavioral change point analysis to identify behavioral states of golden eagles (*Aquila* *chrysaetos*) in the Sonoran and Mojave Deserts of the southwestern United States, and we identify, for each behavioral state, conservation‐relevant habitat associations.
3. We modeled behavior using 186,859 GPS points from 48 eagles and identified 2,851 distinct segments comprising four behavioral states. Altitude above ground level (AGL) best differentiated behavioral states, with two clusters of short‐distance movement behaviors characterized by low AGL (state 1 AGL = 14 m (median); state 2 AGL = 11 m) and two associated with longer‐distance movement behaviors and characterized by higher AGL (state 3 AGL = 108 m; state 4 AGL = 450 m).
4. Behaviors such as perching and low‐altitude hunting were associated with short‐distance movements in updraft‐poor environments, at higher elevations, and over steeper and more north‐facing terrain. In contrast, medium‐distance movements such as hunting and transiting were over gentle and south‐facing slopes. Long‐distance transiting occurred over the desert habitats that generate the best updraft.
5. This information can guide management of this species, and our approach provides a template for behavior‐specific habitat associations for other species of management concern.

## Graphical abstract

Despite the importance of linking habitat use to specific behaviors, this is almost never done, in part because of the many challenges in understanding where and when individual behaviors occur. Here, we use long‐term, short‐interval, and highly precise GPS data as input to recently developed models to interpret animal behavior from telemetry data to identify where and when behavioral states occur. Subsequently, we link these behavioral states to habitat, as a mechanism to identify the environmental correlates of each behavior, and to infer potential threats to these animals based on those behavior‐specific habitat associations.

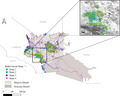

## 1 INTRODUCTION

A central theme underpinning conservation is the need to understand how animals use, and are affected by change in, the landscapes they inhabit (Baldwin et al., 2018; Betts et al., 2019). Study of this problem frequently involves choosing a set of habitat characteristics and relating these to patterns in species occurrence or abundance (Johnson, 1980; Thurfjell et al., 2014). However, occurrence and abundance do not fully capture the effect of habitat on demography and behavior. Landscapes are typically not uniformly used, and, in fact, there is good evidence that habitat use, and consequently anthropogenic effects on wildlife, vary with time, by age classes, or even across behavioral states (Miller et al., 2017; Perona et al., 2019; Zeller et al., 2019). Although management actions sometimes account for variation in habitat associations with time of year and individual age, developing conservation schemes for habitat use specific to different behavioral states presents a unique set of challenges (e.g., if foraging only occurs in one habitat type, then protection of key prey species may be less useful in other habitats). In fact, one of the key reasons that conservation programs are rarely targeted at specific behaviors is because of the difficulties in understanding where and when individual behaviors occur.

Golden eagles (*Aquila* *chrysaetos*) are of interest for conservation in North America as their populations face a number of challenges, including risk from illegal shooting, electrocution, lead poisoning, and collision (USFWS, 2016). Conservation of a viable population of golden eagles requires integrated planning and management at the landscape level. This is especially true because the birds are thought to use different habitats for behaviors such as breeding, foraging, and transiting. However, as has been the case for so many other species, there have been few opportunities to quantify these expectations concerning differential habitat use, and this gap can limit options for conservation management.

Recent advances in the field of movement ecology present opportunities to characterize behavioral states of animals using high‐resolution telemetry data (Kays et al., 2015). A commonly used method of characterizing telemetry data to identify behavior is to employ thresholds and filters to identify movement tracks associated with specific behavior types (Edelhoff et al., 2016). When this is done, thresholds are often defined using expert knowledge and observations in the field. A newer approach involves use of derived movement attributes, such as speed and turning angle, together with modern statistical models (e.g., state space, maximum entropy, Gaussian mixture, exponential‐segment mixture), to define behavioral states (Tracey et al., 2013; Zhang et al., 2015). However, many of these models are difficult to implement and computationally challenging.

Behavioral change point analyses (BCPA) and clustering present new opportunities for ecology and conservation because, like the complex models noted above, they interpret derived movement attributes, but they are much more straightforward to implement and are computationally efficient (Gurarie et al., 2009). BCPA is a likelihood‐based method that detects structural changes in movement parameters that correspond to shifts in behavior. In addition to its ease of implementation, BCPA models are robust to data gaps and to measurement errors that are common in telemetry data (Gurarie et al., 2016). That said, BCPA is still fairly new, and we know of no study in which behavioral states identified with this tool have subsequently been associated with habitat types.

Here we use BCPA to identify behavioral states of golden eagles in the Sonoran and Mojave Deserts of the southwestern United States, and we then use this knowledge to understand, for each behavioral state, habitat associations relevant to species conservation. In these deserts, Golden Eagles encounter threats from climate change and renewable energy development (Braham et al., 2015; Vandergast et al., 2013) and there is management interest in understanding how eagle behavior may influence their vulnerability to renewable energy development. To address this information need, we used a BCPA and clustering to identify eagle behaviors, and then, subsequently, we asked if different behaviors occurred with equal frequencies in different habitat types. To our knowledge, attribute‐based movement analyses have not previously been used to meet these types of ecological and conservation research goals. As such, this analytical approach provides unique information that can augment recently implemented conservation strategies in the study area (CBI, 2013).

## 2 METHODS

### 2.1 Study area

**FIGURE 1:**
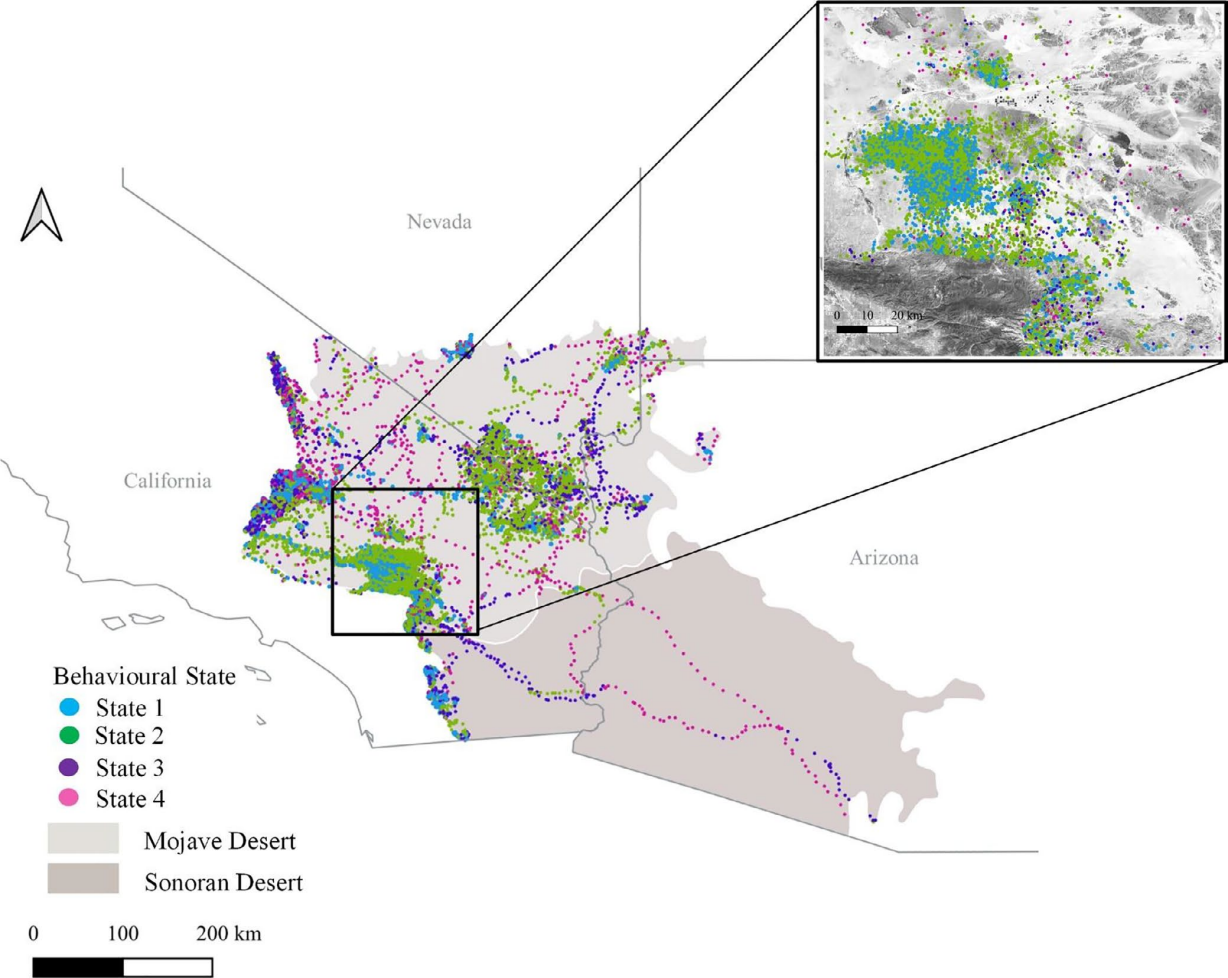
Map of the study area including the Mojave and Sonoran Deserts within the USA. This includes the area covered by the Desert Renewable Energy Conservation Plan (DRECP) for California. Map also shows the GPS locations, colored by behavioral state, of 48 golden eagles tracked from 2012 to 2017 within the Mojave and Sonoran Deserts within the USA. Inset shows how state 1 is in clusters in steeper terrain, whereas state 2 is more dispersed and often over flatter terrain

We collected GPS telemetry data from golden eagles inhabiting or transiting through the Mojave and Sonoran Deserts of the United States during the period 2012–2017 (Figure 1). Both regions have a dry subtropical desert climate with hot summers and warm winters supporting shrub communities with visually dominant succulents or trees (Turner, 1994). Some desert nesting eagles spend time uphill, in areas with higher elevations and different climates (Braham et al., 2015), but we focused analysis on data collected from desert elevations and climate. The Mojave Desert is characterized by creosote bush (*Larrea tridentata*), white bursage (*Ambrosia* *dumosa*), and various visually dominant yuccas (e.g., Joshua tree*—Yucca brevifolia* or *Y*. *jaegeriana*), while the Sonoran Desert supports creosote bush, triangle‐leaf bursage (*Ambrosia* *deltoidea*), and palo verde (*Parkinsonia microphyllum*, and *P*. *floridum*), with giant saguaro cactus (*Carnegiea gigantea*) more prevalent in the Arizona Upland Subdivision of the Sonoran Desert (Turner & Brown, 1982). Golden eagles are sparsely distributed in these regions, nesting mostly on cliffs, in rugged areas adjacent to the broad slopes that support thermal generation, eagle foraging, and renewable energy development (Latta & Thelander, 2013).

### 2.2 Telemetry data collection

Golden eagles were trapped using bow net traps set over carcasses (Bloom et al., 2007) or by hand in the nest, and each bird outfitted with 80–95 g solar‐powered GPS/GSM (Global Positioning System/Global System for Mobile Communications) transmitters (Cellular Tracking Technologies). Telemetry units were attached as backpacks with a nonabrasive Teflon ribbon harness (Kenward, 1985). Each free‐flying bird was aged using molt patterns (Bloom & Clark, 2001; Jollie, 1947) as preadult (hatch‐year, 2Y, 3Y, or 4Y of age) or adult (>4Y), with sex identified based on morphology (Bortolotti, 1984; Edwards & Kochert, 1986; Watson, 2010) and, in most cases, confirmed genetically (Doyle et al., 2014). The telemetry units collected information on GPS locations, fix quality (2D or 3D), and horizontal and vertical dilution of precision (HDOP and VDOP). Data were recorded at intervals of either 15 min or 30 s, stored on the units, and then uploaded to the internet through GSM networks.

### 2.3 Data processing and associations

We standardized fix interval by subsampling 30 s data to 15‐min intervals. Telemetry data were processed to remove 2D fixes and fixes where HDOP or VDOP ≥10 (D’eon and Delparte, 2005; Poessel et al., 2016). We removed GPS data collected before dawn and after dusk, as defined by civil twilight (R package ‘maptools’ v 9‐4; Bivand & Lewin‐Koh, 2018). We also removed data collected from hatch‐year birds prior to their independence from their parents. We considered the birds to be independent once they had moved >10 km from their natal nest for the first time.

We used ArcGIS 10.3.1 to associate each GPS location from an eagle with topographical data on elevation, slope, aspect, topographical position index (TPI), and terrain ruggedness index (TRI), all measured at 30‐m resolution (USGS, 2015). Because aspect data are a circular measure in degrees, for analysis we converted them into two Euclidean vectors, termed eastness (positive values face east) and northness (positive values face north; Roberts, 1986). TPI was estimated with Topography Tools for ArcGIS (Dilts, 2015) and was classified into one of four landform categories, canyons, steep slopes, gentle slopes, or ridges (Jenness et al., 2013). TRI was estimated with Geomorphometry and Gradient Metrics Tools (Evans et al., 2014; Riley et al., 1999) as a continuous variable. We also associated GPS locations with 30‐m land cover data from National Gap Analysis Program (USGS GAP, 2011). We condensed these land cover data into four categories, semidesert, forest, rock vegetation, and shrubland and grassland. Semidesert was the most widespread land cover type in our study and included vegetation dominated by xeromorphic growth forms that varied from shrub‐scrub to complexes of succulents, thornscrub, and microphyllous‐leaved subshrubs (USGS GAP, 2011). Forests included tropical, temperate deciduous, and coniferous forest types. Rock vegetation included a variety of near barren landscapes to desert pavement, rocky slopes, and cliffs sparsely vegetated by vascular plants. Finally, shrubland and grassland communities mostly included mesomorphic perennial grasses and shrubs, with smaller quantities of perennial forbs. We did not include a predictor describing urban areas, as golden eagles avoid these areas but their response is likely more fine‐scale than that measured by the habitat maps (Katzner et al., 2021).

We calculated altitude above ground level (AGL) for each GPS location as the difference between altitude above sea level as measured by the telemetry unit and the estimate of ground elevation at that point obtained from a 30‐m resolution digital elevation model (USGS, 2015). Next, we filtered out GPS locations for which AGL values were < −50 m and >4,000 m (Katzner et al., 2012; Poessel et al., 2018). To assess correlation among predictor variables, we calculated bivariate Pearson correlation coefficients for all pairs of topographic covariates. If variables were either positively or negatively correlated to each other (|*r*| > .55; a conservative threshold; Dormann et al., 2013), we retained for analysis only the one variable of the pair that we thought would provide the more logical biological insight.

### 2.4 Analysis

We applied a two‐step process to classify distinct behavioral states indicated by the telemetry data (Zhang et al., 2015). These steps were (a) segmentation: a process to detect significant change or break points in a trajectory based on a selected movement parameter and (b) clustering: a process to identify the groups of segments that have similar characteristics, and thus likely indicative of similar movement behavior. Once we had clustered similar segments together, then we used statistical tools to model differences in habitat association for each behavioral cluster.

#### 2.4.1 Segmentation

We used filtered eagle locations as input into a behavioral change point analysis (function ‘windowsweep’, R package ‘bcpa’ v 1.1; Gurarie, 2014; Gurarie et al., 2009) to partition, for each bird, series of locations into discrete segments separated by changes in trajectory. We used persistence velocity as the movement parameter to input into the BCPA model. Persistence velocity captures the tendency and magnitude of a movement to persist in a given direction and is calculated as the product of instantaneous speed and cosine of the turning angle (Gurarie et al., 2009; Teimouri et al., 2018; Zhang et al., 2015). The BCPA identifies changes in persistence velocity values by using likelihood comparisons in a moving window over the time series of those values. We used a window size of 30 sequential locations and, within the window, located the most likely change point using the Bayesian information criterion (Gurarie et al., 2009). We also at tested if changes in settings of parameters such as the size of the window had an impact on the number of segments detected. Mathematical details of the calculation of persistence velocity and the formulation of BCPA can be found in Gurarie et al. (2009). The output from the BCPA provides information on change points needed to distinguish the distinct behavioral segments that we then input into the clustering analysis.

#### 2.4.2 Clustering

We grouped the segments identified by the BCPA using a *k*‐mean cluster analysis. To do this, we first calculated, for each segment, the median of the instantaneous speed (in km/hr), the relative turning angle, and the AGL (Zhang et al., 2015). We input these characteristics into our clustering algorithm, and we selected the optimal number of clusters using the ‘elbow method’ (function ‘fviz_nbclust’, method = “wss”, R package NbClust v 3.0; Charrad et al., 2014). Because the cluster analyses do not account for bird‐to‐bird variability (i.e., it is not possible to include a random effect for bird in this analysis), we first identified an optimal number of clusters for each bird separately and subsequently on data for all birds combined together. Since the optimal number of clusters for individual birds was similar, we then grouped segments into that optimal number of clusters using data for all birds together (function ‘kmeans’, R packages ‘stats’ v 3.4.3 and ‘fpc’ v 2.1‐ 11.1). We used a Wilcoxon–Mann–Whitney test to evaluate parameter differences among states identified by the BCPA.

#### 2.4.3 Statistical analysis

Once we identified clusters, we modeled cluster‐(i.e., behavioral state‐) specific response of eagles to topographic and land cover features. We wished to use a multinomial model for this analysis. However, we could find no modeling tool that accurately incorporated >1 random effect into a computationally efficient multinomial analysis, as required of this analysis (see below). Instead, we created a set of generalized mixed effect logistic regression models for pairs of state‐specific responses (function ‘glmer’, R package ‘lme4’ 1.1‐15; Dobson & Barnett, 2018 provide a justification for this approach).

In each model, the response variable described the probability of being in one of two behavioral states that were identified by clustering algorithms. After removing correlated variables (Table S1), fixed effects in these models were the continuous elevation, northness, eastness, and slope variables, and the categorical bird age, land cover, and TPI variables. These models had a logit link function and, because we were not interested to test the effect of these two variables, random effects for month (12 months) and bird ID (54 birds). When building our dataset for modeling, we increased the age of eagles each January. We also rescaled all continuous variables by subtracting the mean and dividing by twice the standard deviation (Gelman, 2008).

We evaluated competing models in an information theoretic framework, and when a single model did not account for the majority of model weights, we averaged parameter estimates from models with weights ≥0.01 (functions ‘dredge’ and ‘model.avg’, R package ‘MuMIn’ 1.43.1, Anderson, 2007; Barton, 2018; Anderson and Burnham, 2002). For comparative purposes, we also ran an identically parameterized multinomial model but with a single random effect for bird ID (omitting random effects for month and bird age; R package ‘mclogit’, Elff, 2018). We used a baseline category logit model with state 1 (described in results) as the baseline category.

## 3 RESULTS

We tracked 10–20 eagles each year from 2012 to 2017, resulting in 955,462 GPS locations collected from 21 male and 33 female eagles. Of these 54 eagles, 31 were preadults for the entire study, 12 were adults for the entire study, and 11 were preadults at capture but became adults during the course of the study. After subsampling and filtering, we considered as input into BCPA models 186,859 data points from 48 birds (19 males, 29 females, 25 preadults, 12 adults, and 11 whose age class changed during the course of the study; Table S2), for a total of 81 bird‐years.

### 3.1 Identification of behavioral states

We identified 2,851 distinct segments within the eagle telemetry data. Regardless of whether we ran these analyses with all birds grouped together or separately for each bird, the optimal number of grouping clusters (i.e., behavioral states) for these segments was 4 (average “silhouette width” for all birds = 0.487; Figures S1 and S2). We refer to these four behavioral states as states 1–4 (Table 1). In the analysis for all birds, states 1 and 2 were the most commonly observed, making up 58% and 39% of data points, respectively (Table 1; Figure S3). States 3 (2%) and 4 (1%) were less commonly observed. Because the four states in this model were not identical to the four states in the models for each bird, not all birds were represented in all states (Table 1).

**TABLE 1:** Summary statistics (mean ± *SE*) for the four behavioral states identified by a behavioral change point analysis of GPS telemetry data gathered from golden eagles in the Mojave and Sonoran Desert of the southwestern USA. Summary statistics are for number of data points and of birds, altitude above ground level (AGL), instantaneous movement speed, and turning angle of the trajectory. See text for description of how each behavioral state was defined

Behavior	*n* points	*n* birds	AGL (m)	Speed (km/hr)	Turn angle (deg.)
State 1 – low altitude	108,795	47	52 ± 5	8 ± 1	131 ± 2
State 2 – low altitude	72,930	44	69 ± 6	11 ± 1	115 ± 2
State 3 – high altitude	3,502	28	293 ± 29	56 ± 12	96 ± 7
State 4 – high altitude	1,632	21	545 ± 53	105 ± 27	76 ± 9

**FIGURE 2:**
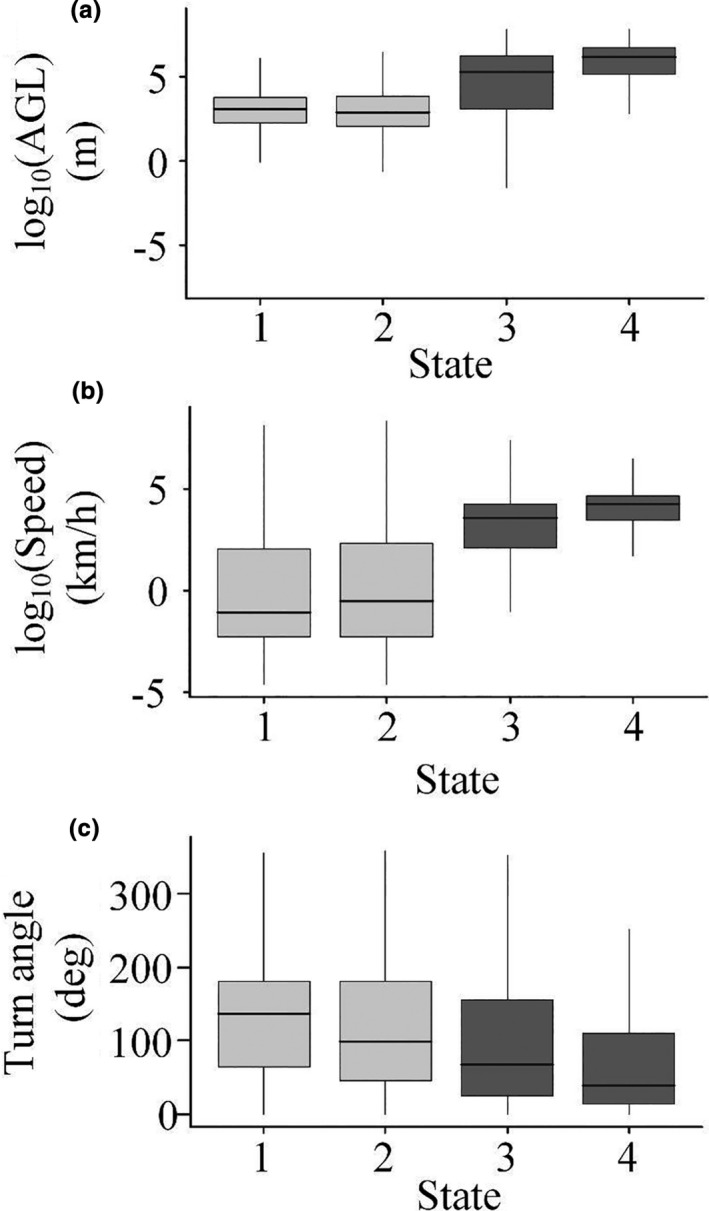
Boxplot of (a) altitude above ground (AGL), (b) speed, and ​(c) turning angle of the four behavioral states identified by BCPA (behavior change point analysis). AGL and speed were log‐transformed more clearly illustrate variation among groups. Light gray boxplots represent the two low‐altitude states (state 1 and 2), and the dark gray boxplots represent the high‐altitude states (state 3 and 4). Lower and upper box boundaries represent 25th and 75th percentiles, respectively, and line inside the box represents the median

Altitude above ground level was the best differentiator among behavioral states (Table 1, Figure 2a), with two clusters characterized by low AGL (state 1 AGL = 14 m (median); state 2 AGL = 11 m; for means, see Table 1) and two by higher AGL (state 3 AGL = 108 m; state 4 AGL = 450 m) (Wilcoxon–Mann–Whitney test for AGL of states 1 & 2 vs. states 3 & 4: *z* = 60.14, *p* <.001). Behavioral states that occurred at lower altitudes also had distinctly lower speed (*z* = 79.80, *p* <.001; Table 1, Figure 2b) and somewhat higher turning angles (*z* = −32.36, *p* <.001; Table 1, Figure 2c) than did states at higher altitudes.

The two low‐altitude behavioral states also were well separated by flight altitude, with state 1 generally occurring at the lowest altitudes (Table 1) (*z* = 23.966, *p* < .001). Despite the fact that flight speeds were similar between the two classes (*z* = 0.72997, *p* = .4654), turn angles were greater in state 1 than in state 2 (*z* = 84.368, *p* < .001). The two high‐altitude behaviors were also strongly differentiated by flight altitude (*z* = −38.08, *p* < .001) and velocity (*z* = −39.32, *p* < .001), with state 4 characterized by behaviors that were higher, faster, and with more variation in speed than those in state 3 (Table 1). Turn angles also tended to be greater in state 3 than in state 4 (*z* = 20.198, *p* < .001).

### 3.2 Environmental correlates of variation in behavioral states

Correlation analysis showed that two of our potential habitat predictors, TRI and slope, were highly correlated (Table S1). We removed TRI from our models describing behavioral associations with habitat because it is a derived variable and we believed that the slope variable provided a more intuitive interpretation. Because we observed that AGL so strongly separated behaviors into two groups, our first logistic regression described the probability of being in a high‐altitude state (states 3 and 4 together) versus that of being in a low‐altitude state (states 1 and 2 together), given the habitat predictors. Subsequently, we ran similar models describing the probability of being in one low‐altitude state versus the other (state 1 vs. state 2) and of being in one high‐altitude state versus the other (state 3 vs. state 4), in each case given the habitat predictors.

**FIGURE 3:**
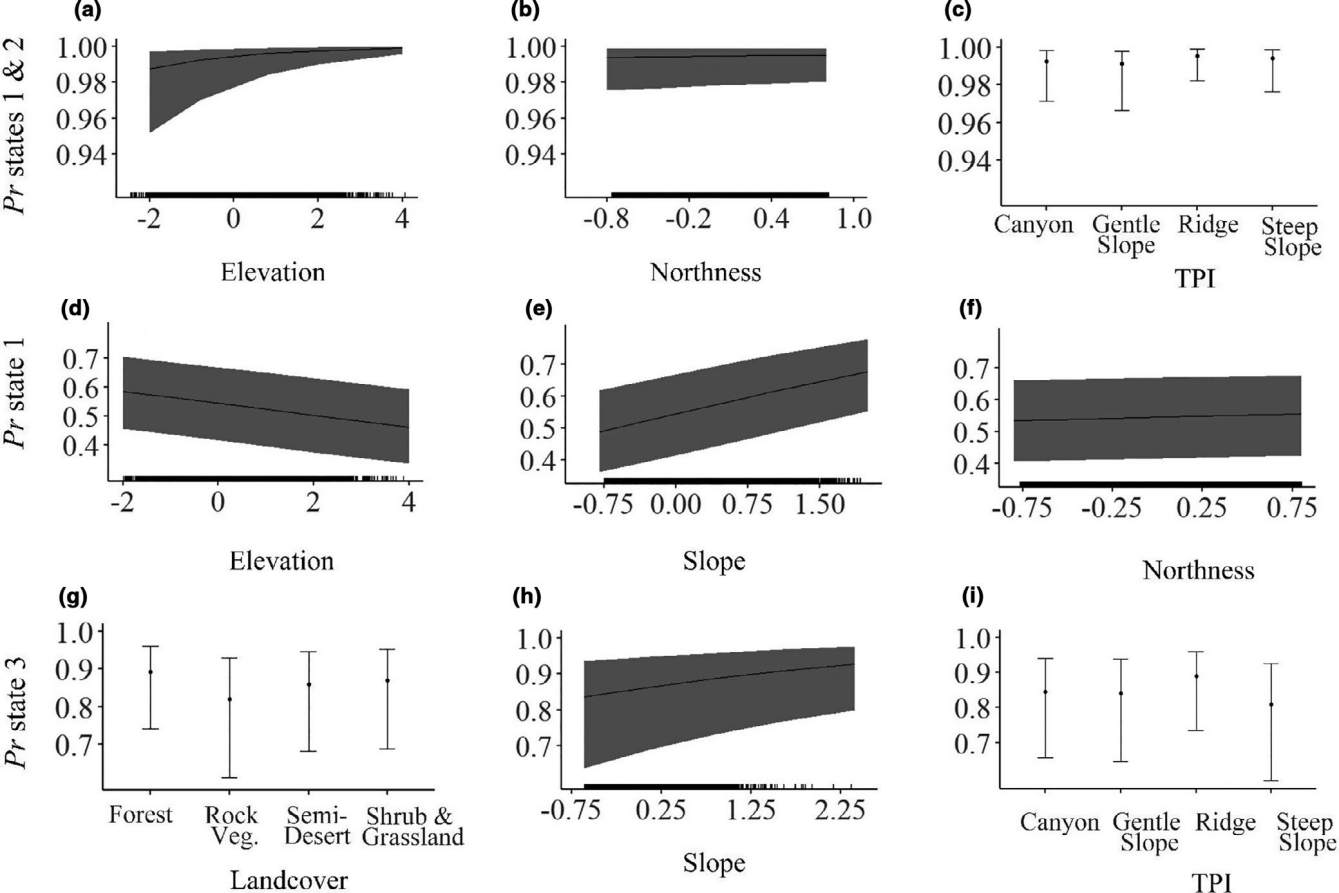
Plots describing the probability of golden eagles tracked by telemetry in the Mojave and Sonoran Deserts, 2012–2017, of being in one behavioral state versus another. Shown are, for each of three model sets, plots of the top three predictors in the best performing model. In our first model, we evaluated the probability of being in a low‐altitude state (states 1 or 2, as opposed to being in a high‐altitude state, states 3 or 4) as a function of (a) ground elevation, (b) northness of slope, and (c) Topographic Position Index (TPI) of the terrain. In our second model, we evaluated the probability of being in one of two low‐altitude states (state 1 vs. 2; reference level state 2), as a function of (d) ground elevation, (e) degree of slope, and (f) northness. In our third model, we evaluated the probability of being in one of two high‐altitude states (state 3 vs. 4; reference level state 4), as a function of (g) land cover, (h) degree of slope, and (i) TPI

There were strong differences in habitat at locations where eagles were in low (states 1 and 2) versus high (states 3 and 4) altitude behavioral states. The top model describing response of state to habitat had 99% of model weights and included fixed effects for all model parameters (Table 2a). Low‐altitude behavioral states were more likely to occur over higher ground elevations, on ridges, steep slopes, and canyons, and on slopes that faced in more northerly and westerly directions (Figure 3a–c, Table 3). They were less likely to occur on gently sloping terrain. Although inclusion of land cover improved the fit of our model, we did not detect an effect of land cover class on the probability of being in a low‐ or high‐altitude behavioral state (Table 3). Finally, younger birds were more likely to fly at lower altitudes.

**TABLE 2:** Results of the top five models describing factors affecting probability of being in (a) low altitudes (state 1 and state 2) compared to high altitudes of golden eagles (state 3 and state 4); (b) one of the two low‐altitude states (state 1 vs. state 2); and (c) one of the two high‐altitude states (state 3 vs. state 4) in the Mojave and Sonoran Deserts, 2012–2017. We used logistic regression to model eagle response, with bird ID, bird age, and month of the year as random effects and predictors as described below and in the main text

Comparison	Predictors in top 5 models in set	AIC_c_	ΔAIC_c_	*w*_i_
(a) 1 & 2 vs. 3 & 4	Elevation + Eastness + Northness + TPI + Slope + Land cover + Age	26,765	0	0.99
	Elevation + Eastness + Northness + TPI + Slope + Age	26,775	10.5	0.01
	Elevation + Northness + TPI + Slope + Land cover + Age	26,776	11.1	0.00
	Elevation + Eastness + Northness + TPI + Land cover + Age	26,778	12.9	0.00
	Elevation + Eastness + TPI + Slope + Land cover + Age	26,780	15.8	0.00
(b) 1 vs. 2	Elevation + Northness + TPI + Slope + Land cover + Age	205,322	0	0.68
	Elevation + Eastness + Northness + TPI + Slope + Land cover + Age	205,324	1.5	0.32
	Elevation + Northness + Slope + Land cover + Age	205,336	13.4	0.00
	Elevation + Eastness + Northness +Slope + Land cover + Age	205,337	15.0	0.00
	Elevation + TPI + Slope + Land cover + Age	205,339	16.5	0.00
(c) 3 vs. 4	Northness + TPI + Slope + Land cover	5,246	0	0.15
	Eastness + Northness + TPI + Slope + Land cover	5,247	0.4	0.12
	Northness + TPI + Slope + Land cover	5,248	0.8	0.10
	Elevation + Northness + TPI + Slope + Land cover	5,248	1.2	0.08
	Eastness + TPI + Slope + Land cover	5,248	1.6	0.08

**TABLE 3:** Effect estimates for the top model in Table 2a showing probability of being in a low‐altitude state (state 1 and 2) compared to the probability of being in a high‐altitude state (state 3 and 4) as a function of habitat‐related predictors for golden eagles tracked with GPS telemetry between 2012 and 2017 in the Mojave and Sonoran Deserts within the USA. TPI = topographic position index. The reference category for TPI = canyons, for land cover = forest, and for age = adult

Variable	Estimate	*SE*	*z*	*p*
Intercept	4.38	0.70	6.26	<.001
Elevation	0.40	0.03	13.66	<.001
TPI: Ridge	0.48	0.04	10.95	<.001
Age: Preadult	0.91	0.12	7.39	<.001
Northness	0.14	0.03	4.23	<.001
Slope	0.19	0.05	3.87	<.001
Eastness	−0.12	0.03	−3.62	<.001
TPI: Steep Slope	0.20	0.06	3.11	.002
TPI: Gentle Slope	−0.16	0.06	−2.64	.008
Land cover: Semidesert	−0.09	0.06	−1.55	.121
Land cover: Rock Vegetation	0.09	0.06	1.39	.164
Land cover: Shrubland & Grassland	−0.02	0.09	−0.23	.819

The two low‐altitude behavioral states (1 and 2) also did not occur in the same habitats (Table 2b). In this case, the top two models together accounted for >99% of model weights. These models only differed by inclusion of an eastness parameter. The best differentiators of the two behaviors were slope and elevation, with the probability of being in state 1 increasing with slope and decreasing with elevation (Figure 1 inset, Figure 3d,e, Table 4). Eagles also were more likely to be in state 1 with increasing northness of slope (Figure 3f), in semidesert habitats, and over steep slopes and ridges. They were less likely to be in this state over shrubland and grasslands than over forests. Finally, there was a strong age effect, with young birds more likely to be in state 1 than in state 2.

**TABLE 4:** Averaged effect estimates for the top two models from Table 2b showing probability of being in one of two low‐altitude states (state 1 vs. state 2) as a function of habitat‐related predictors for golden eagles tracked with GPS telemetry between 2012 and 2017 in the Mojave and Sonoran Deserts within the USA. TPI = topographic position index. The reference category for TPI = canyons, for land cover = forest, and for age = adult

Variable	Estimate	Adjusted *SE*	*z*	*p*
(Intercept)	−0.34	0.27	1.22	.223
Age: Preadult	0.78	0.02	49.61	<.001
Slope	0.28	0.02	17.70	<.001
Elevation	−0.08	0.02	5.38	<.001
Northness	0.05	0.01	4.28	<.001
Land cover: Shrubland & Grassland	−0.16	0.04	3.88	<.001
Land cover: Semidesert	0.11	0.03	3.66	<.001
TPI: Steep Slope	0.05	0.02	2.33	.020
TPI: Ridge	0.03	0.02	1.79	.073
Land cover: Rock Vegetation	0.03	0.03	1.10	.274
Eastness	0.01	0.01	0.69	.492
TPI: Gentle Slope	−0.01	0.02	0.28	.783

The two high‐altitude behavioral states (3 and 4) also occurred over different habitat types (Table 2c). In this case, the top three models only accounted for 37% of model weights. Model averaging suggested that the locations where these two behaviors occurred were differentiated by substantially different features than those that differentiated the two low‐altitude states. In this case, golden eagles were less likely to be in state 3 over rocky vegetation, but more likely to be in that state over steeper and east‐facing slopes, and over TPI categories for ridges (Figure 3g–i, Table 5). In contrast, eagles were more likely to be in state 4 over semidesert, the TPI category for steep slopes, and over north‐ and west‐facing slopes. There were no age‐related differences in use of these two states.

**TABLE 5:** Average effect estimates from the top four models from Table 2c showing probability of being in one of two high‐altitude states (state 3 vs. state 4) as a function of habitat‐related predictors for golden eagles tracked with GPS telemetry between 2012 and 2017 in the Mojave and Sonoran Deserts within the USA. TPI = topographic position index. The reference category for TPI = canyons, for land cover = forest, and for age = adult

Variable	Estimate	Adjusted *SE*	*z*	*p*
(Intercept)	2.52	0.71	3.54	<.001
Land cover: Rock Vegetation	−0.58	0.13	4.28	<.001
TPI: Ridge	0.37	0.09	4.10	<.001
Slope	0.29	0.10	2.83	<.001
Land cover: Semidesert	−0.29	0.12	2.31	.021
TPI: Steep Slope	−0.26	0.13	1.98	.047
Northness	−0.12	0.07	1.67	.095
Eastness	0.09	0.07	1.26	.209
Land cover: Shrubland & Grassland	−0.20	0.20	1.01	.310
Elevation	0.09	0.10	0.94	.345
TPI: Gentle Slope	−0.04	0.11	0.34	.738
Age: Preadult	0.12	0.90	0.13	.893

The results of the multinomial model showed roughly similar results as did the sets of logistic models (Table S3). In this analysis, we used state 1 as our reference because, from a conservation perspective, it was especially important for us to know the specific habitat types for low‐altitude behaviors, such foraging and perching, of eagles. Because the logistic models incorporated multiple random effects that we know were biologically relevant, and because they allowed for more interesting comparisons, we interpret those models to provide ecological and conservation insights.

## 4 DISCUSSION

Our analytical approach is unusual for conservation in that it links specific behaviors to differential habitat use. The process of interpreting these statistically defined behavioral states and subsequently associating them with the habitats in which they occur also leads us to substantive insight into eagle ecology. That insight leads to direct guidance for mitigation or management that could be implemented for golden eagles facing habitat loss in the Sonoran and Mojave Deserts of the American southwest.

### 4.1 Identifying behavioral states of eagles

The descriptive statistics (Table 1, Figure 2) provide a foundation for interpretation and identification of the behavioral states we identified. We interpret the two behavioral states occurring at low altitudes as different types of short‐distance movement behaviors. For example, state 1, which is lower and slower, is likely indicative predominantly of perching (GPS points from a stationary receiver are rarely in exactly the same spot and thus a high turn angle can be characteristic of a nonmoving GPS unit). However, this state likely also included other slow, low, and altitudinally varying flights such as territorial displays, low‐altitude hunting, and approach and departure from nests (Watson, 2010). State 2, which is also low, but less variable in altitude, was faster and more direct, and likely is indicative of transiting within a home range or use area, or of other behaviors such as hunting (Watson, 2010; Wiens et al., 2017). Furthermore, the spatial relationships of these points are consistent with these interpretations. That is, state 1 behaviors occured in clusters in steeper terrain, and state 2 behaviors occured more broadly across the landscape, including flatter terrain (i.e., eagles perch and roost in specific locations but forage more widely around those locations; Figure 1, inset). That these two states are more common than the other two (Table 1) also is consistent with the biology of these birds, many of which spend most of their time within defined home ranges and less time transiting to and from those home ranges (Braham et al., 2015).

We interpret the two behavioral states occurring at high altitudes as indicative of less frequent but longer‐distance transiting behaviors. Transiting by eagles beyond a home range or use area can be characteristic of movements between those ranges or areas (e.g., some territorial eagles make regular movements through the Mojave Desert to high‐elevation foraging areas near Tehachapi California; Braham et al., 2015) or of dispersal (some of the data we considered were from hatch‐year birds that moved southeast through the Sonoran Desert; Figure 1). Because these transiting behaviors are higher, faster, and more direct than behaviors in states 1 or 2, they likely corresponded to two forms of this latter type of longer‐distance transiting behavior. The fact that these two behaviors occurred less frequently than the first two, and the relatively greater proportion of these migration‐like behaviors that occurred in spring and fall (Figure S3), supports this explanation. Furthermore, these expectations corresponded with the spatial arrangement of these points, such that the long‐distance movements appear to be combinations of state 4 (long, fast, direct flights) and state 3 (at the start and end of those long flights) that occur between clusters of movements in states 1 and 2 (Figure 1).

### 4.2 Associating behavioral states with habitat

Our computational approach of linking statistical states to habitat features illustrates that certain behaviors were more likely than others to occur in certain habitats, and this approach provides new conservation‐relevant insight into the drivers of those behaviors. For example, perching, low‐altitude hunting, and territorial behaviors were more likely to occur in environments associated with poor thermal generation, at higher elevations, and over steeper, and more north‐facing terrain. These habitats are those where eagles nest, roost, and use orographic soaring to hunt for prey. In contrast, higher‐altitude hunting behavior and short‐distance transiting were in more gentle and south‐facing slopes. These are areas where thermal generation is improved yet not optimal, and transition zones between flat and steep slopes where prey may be abundant. Finally, the longer‐distance traveling we observed occurred over desert habitats that generate the best updraft but possess lower prey and nesting resources for eagles (i.e., low elevation, south‐facing, gentler or flat terrain; Dunk et al., 2019; Wiens et al., 2017). Because this analysis links a behavioral response by eagles to the topographic environment they encounter, it has important implications for conservation.

### 4.3 Conservation implications

Although conservation relies, in part, on linking species to the habitat they occupy, highly motile species almost never use their habitats uniformly. As such, there is a strong conservation imperative to connect behavioral states to habitat. For example, our study was initially developed at the request of management agencies who were concerned about loss of habitat for golden eagles in the Sonoran and Mojave Deserts (Braham et al., 2015). Past work has suggested that urban development has pushed golden eagles out of historically suitable lower‐elevation nesting habitats in southern California (Bloom, 1991; Scott, 1985).

Our analysis adds considerable nuance to these prior suggestions that help to characterize the habitat associations of the eagles that remain outside of those historically occupied lower‐elevation sites. Behavioral analyses we present here suggest that modern golden eagles engage in behaviors such as foraging and perching in areas at higher elevations and with steeper terrain. There, eagles flew at low altitude above ground and were likely thus strongly influenced by microfeatures of that terrain. In contrast, flatter, lower‐elevation habitats were sometimes used for low‐altitude behaviors such as hunting, but were more likely to be used for transiting flight at higher altitude above ground. When making these longer‐distance, higher‐altitude movements, eagles were less likely to be affected by small scale perturbations to the landscape below (that said, the shape of our data distributions illustrates that some of these movements still occurred at flight altitudes where eagles could encounter risk in those landscapes; Figure 2).

Although urban development is certainly an impediment to eagles (Domenech et al., 2015), our results suggest that smaller scale development that does not dramatically change the updraft environment of flatter, lower‐elevation terrain is unlikely to substantially influence movement behavior of extant eagle populations. Since most solar and many wind energy projects utilize these flatter, lower‐elevation sites (Arnette & Zobel, 2011; Doljak & Stanojević, 2017), our results suggest potential for compatibility between extant eagle populations and carefully sited renewable energy developments in the Mojave and Sonoran Deserts. That said, larger‐scale projects, or those in habitat types eagles use more frequently, may be more impactful to their daily life (Tracey et al., 2014).

## 5 CONCLUSIONS

Eagles have more than four types of behaviors, each of which has conservation relevance. However, by using readily available analytical tools to devolve the diversity of eagle behavior into four states, we were able to simplify a complex problem. Doing so allowed us to identify otherwise obscure and behavior‐specific habitat associations of a high‐priority species that is the focus of extensive conservation management. The approach we used*—*identifying and clustering behavioral states and subsequently linking them to the habitat in which they occurred*—*allowed us to illustrate a small part of the complexity and habitat associations of this species’ behavior. A logical next step for this research would be to expand on the linkages between behavior and the environment, by linking factors such as prey availability, risk to eagles, and seasonal variability in land cover and prey availability.

## CONFLICT OF INTEREST

The authors declare no conflict of interest.

## AUTHOR CONTRIBUTIONS

**Maitreyi Sur:** Conceptualization (lead); Data curation (supporting); Formal analysis (lead); Investigation (lead); Methodology (lead); Writing‐original draft (lead); Writing‐review & editing (lead). **Brian Woodbridge:** Conceptualization (equal); Funding acquisition (equal); Methodology (equal); Project administration (equal); Resources (equal); Supervision (equal); Writing‐review & editing (equal). **Todd C** **Esque:** Conceptualization (equal); Funding acquisition (equal); Investigation (equal); Methodology (equal); Resources (equal); Supervision (equal); Writing‐review & editing (equal). **James** **Belthoff:** Funding acquisition (equal); Methodology (equal); Project administration (equal); Resources (equal); Software (equal); Supervision (equal); Writing‐review & editing (equal). **Peter Bloom:** Data curation (equal); Resources (equal); Validation (equal); Writing‐review & editing (supporting). **Robert N. Fisher:** Data curation (equal); Formal analysis (supporting); Methodology (supporting); Resources (equal); Supervision (supporting); Writing‐review & editing (equal). **Kathleen M. Longshore:** Resources (equal); Supervision (equal); Writing‐review & editing (equal). **Kenneth E**. **Nussear:** Resources (equal); Supervision (equal); Writing‐review & editing (equal). **Jeff Tracey:** Methodology (equal); Resources (equal); Supervision (equal); Writing‐review & editing (equal). **Melissa A. Braham:** Data curation (equal); Project administration (supporting); Resources (equal); Validation (equal); Visualization (equal); Writing‐review & editing (supporting). **Todd** **Katzner:** Conceptualization (equal); Data curation (equal); Formal analysis (supporting); Funding acquisition (lead); Investigation (equal); Methodology (lead); Project administration (lead); Resources (equal); Software (equal); Supervision (lead); Validation (equal); Visualization (equal); Writing‐original draft (lead); Writing‐review & editing (lead).

## Supporting information

Supplementary Material

## Data Availability

Additional information and figures supporting this article have been uploaded as part of the electronic supplementary material. The underlying code for our analyses is publicly available and cited in our manuscript, as are the associated environmental datasets. However, the golden eagles we study are both protected and persecuted, within the USA and globally. Nest locations are one of the primary sites of persecution of eagles, and the raw biotelemetry data we analyzed show those nest locations. As a consequence, we do not make data publicly available. A representative sample of these data without nest locations have been uploaded to the Boise State University on‐line repository (https://doi.org/10.18122/bio_data.7.boisestate) and data can be made available for research upon request to authors M. Sur (maitreyisur@boisestate.edu).
