# Supplementary material for: Linking behavioral states to landscape features for improved conservation management
Source: Ecol Evol. 2021 May 25;11(12):7905–16. doi: 10.1002/ece3.7621 (PMC8216984; doi:10.1002/ece3.7621)
Supplement: Supplementary file 1 — Supplementary Material [file ECE3-11-7905-s001.docx]

**Supporting Information**

**SI Table 1.** Correlation matrix for topographical variables associated with movement of 48 golden eagles tracked in the Mojave and Sonoran Deserts within the USA, including the area covered by the Desert Renewable Energy Conservation Plan (DRECP) for California. Bold values indicate variables with a correlation >0.55. TPI = topographical position index, TRI = terrain ruggedness index.

|  | Elevation | Northness | Eastness | Slope | TPI | TRI |
| --- | --- | --- | --- | --- | --- | --- |
| Elevation | 1 | 0.05 | 0.1 | 0.42 | 0.22 | 0.4 |
| Northness |  | 1 | 0.07 | 0.14 | -0.03 | 0.12 |
| Eastness |  |  | 1 | 0.06 | 0.03 | 0.05 |
| Slope |  |  |  | 1 | 0.27 | **0.86** |
| TPI |  |  |  |  | 1 | 0.25 |
| TRI |  |  |  |  |  | 1 |

**SI Table 2.** Number of male, female, pre-adult, and adult golden eagles tracked during 2012-2017 to understand factors affecting behavior-specific habitat use in the Mojave and Sonoran Deserts within the USA. Column totals are not sums of the counts in the columns because individuals tracked in >1 year were only counted once to calculate the total. Likewise, 11 birds were captured as pre-adults but tracked into adulthood and thus show up in totals for both the pre-adult and adult categories. Telemetry units tracked golden eagles for an average of 221 days per bird (range = 2 to 2106 days).

| Year | Male | Female | Pre-adult | Adult | Total |
| --- | --- | --- | --- | --- | --- |
| 2012 | 4 | 8 | 12 | 0 | 12 |
| 2013 | 12 | 8 | 9 | 11 | 20 |
| 2014 | 5 | 7 | 6 | 6 | 12 |
| 2015 | 1 | 11 | 8 | 4 | 12 |
| 2016 | 3 | 11 | 7 | 8 | 15 |
| 2017 | 4 | 6 | 5 | 5 | 10 |
| Total | 19 | 29 | 36 | 23 | 48 |

| Model | Variable | Estimate | SE | z | *P* |
| --- | --- | --- | --- | --- | --- |
| **state 1 vs state2** | Intercept | 2260.00 | 30810.00 | 0.07 | 0.942 |
|  | Slope | -0.30 | 0.02 | -19.16 | <0.001 |
|  | Land cover: Semi-Desert | -0.22 | 0.03 | -6.66 | <0.001 |
|  | Eastness | 0.06 | 0.01 | 5.44 | <0.001 |
|  | Land cover: Shrubland & Grassland | 0.21 | 0.04 | 5.15 | <0.001 |
|  | Land cover: Rock Vegetation | -0.13 | 0.03 | -4.02 | <0.001 |
|  | Elevation | 0.06 | 0.02 | 3.71 | <0.001 |
|  | TPI: Gentle Slope | 0.08 | 0.02 | 3.49 | <0.001 |
|  | TPI: Steep Slope | 0.06 | 0.02 | 2.58 | 0.010 |
|  | Northness | -0.02 | 0.01 | -1.45 | 0.146 |
|  | TPI: Ridge | -0.02 | 0.02 | -1.28 | 0.200 |
| **state 1 vs state 3** | Intercept | -201.91 | 689.00 | -0.29 | 0.769 |
|  | Land cover: Semi-Desert | -2.46 | 0.07 | -34.98 | <0.001 |
|  | Land cover: Rock Vegetation | -2.49 | 0.07 | -33.54 | <0.001 |
|  | Elevation | 0.41 | 0.03 | 11.90 | <0.001 |
|  | Land cover: Shrubland & Grassland | -1.13 | 0.10 | -11.36 | <0.001 |
|  | Slope | -0.31 | 0.05 | -6.87 | <0.001 |
|  | TPI: Gentle Slope | 0.36 | 0.06 | 6.14 | <0.001 |
|  | TPI: Ridge | -0.14 | 0.04 | -3.21 | 0.001 |
|  | Northness | -0.04 | 0.03 | -1.15 | 0.249 |
|  | TPI: Steep Slope | 0.06 | 0.07 | 0.98 | 0.329 |
|  | Eastness | 0.03 | 0.03 | 0.92 | 0.358 |
| **state 1 vs state 4** | Intercept | -182.47 | 1.34 | -136.39 | <0.001 |
|  | Land cover: Semi-Desert | -12.82 | 0.10 | -126.50 | <0.001 |
|  | Land cover: Rock Vegetation | -13.33 | 0.11 | -124.89 | <0.001 |
|  | Elevation | 4.28 | 0.05 | 93.86 | <0.001 |
|  | Land cover: Shrubland & Grassland | -4.64 | 0.15 | -30.84 | <0.001 |
|  | TPI: Gentle Slope | 1.27 | 0.08 | 15.56 | <0.001 |
|  | TPI: Ridge | 0.75 | 0.06 | 12.66 | <0.001 |
|  | Eastness | 0.42 | 0.05 | 8.92 | <0.001 |
|  | Slope | 0.29 | 0.06 | 4.59 | <0.001 |
|  | TPI: Steep Slope | -0.20 | 0.09 | -2.30 | 0.022 |
|  | Northness | 0.07 | 0.05 | 1.50 | 0.135 |

**SI Table 3.** Effect estimates from a multinomial model results showing probability of being in low altitude state 1 as a function of habitat-related predictors for golden eagles tracked with GPS telemetry between 2012 and 2017 in the Mojave and Sonoran Deserts within the USA. TPI = topographic position index. The reference category for TPI = canyons, and for land cover = forest.

**SI Figure 1.** Within groups sums of squares used to identify the optimal number of clusters of behavioral models of data from all golden eagles together. Solid black line shows total within cluster sum of squares plotted as a function of number of clusters, calculated using k mean clustering algorithm. The dotted line indicates the optimal number of clusters (*k* = 4) we used in the final clustering analysis.

**
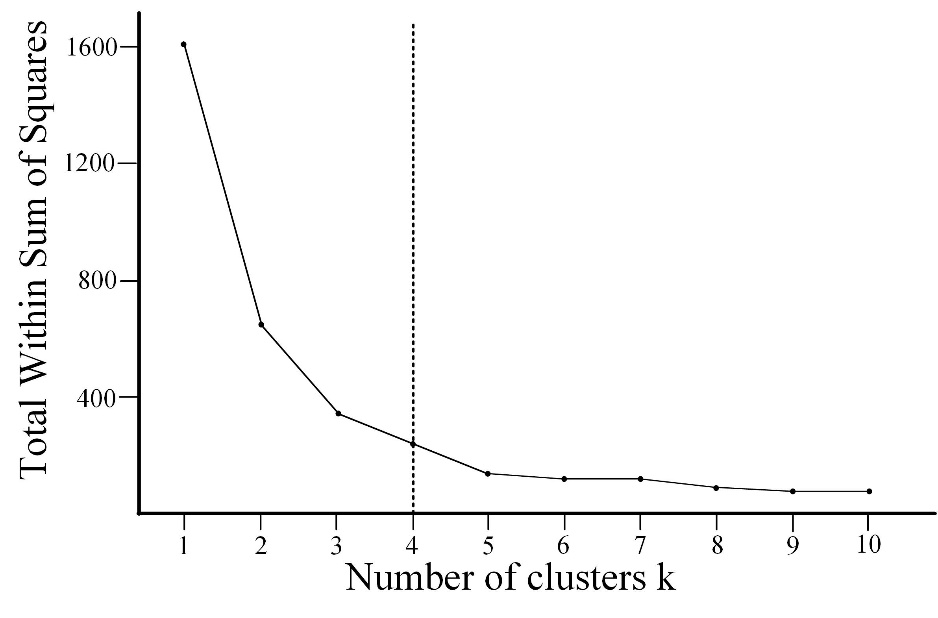
**

**SI Figure 2.** Within groups sums of squares used to identify the optimal number of clusters of behavioral models of data from each individual golden eagle for which >3 clusters could be identified. Solid black line shows total within cluster sum of squares plotted as a function of number of clusters, calculated using k mean clustering algorithm. The dotted line indicates the optimal number of clusters (usually *k* = 4).

**
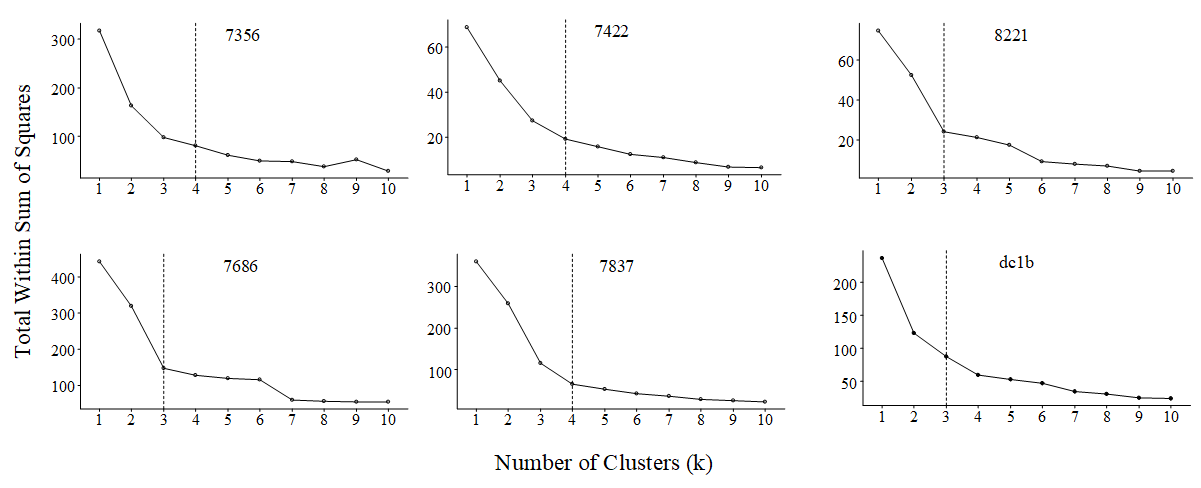
**

**
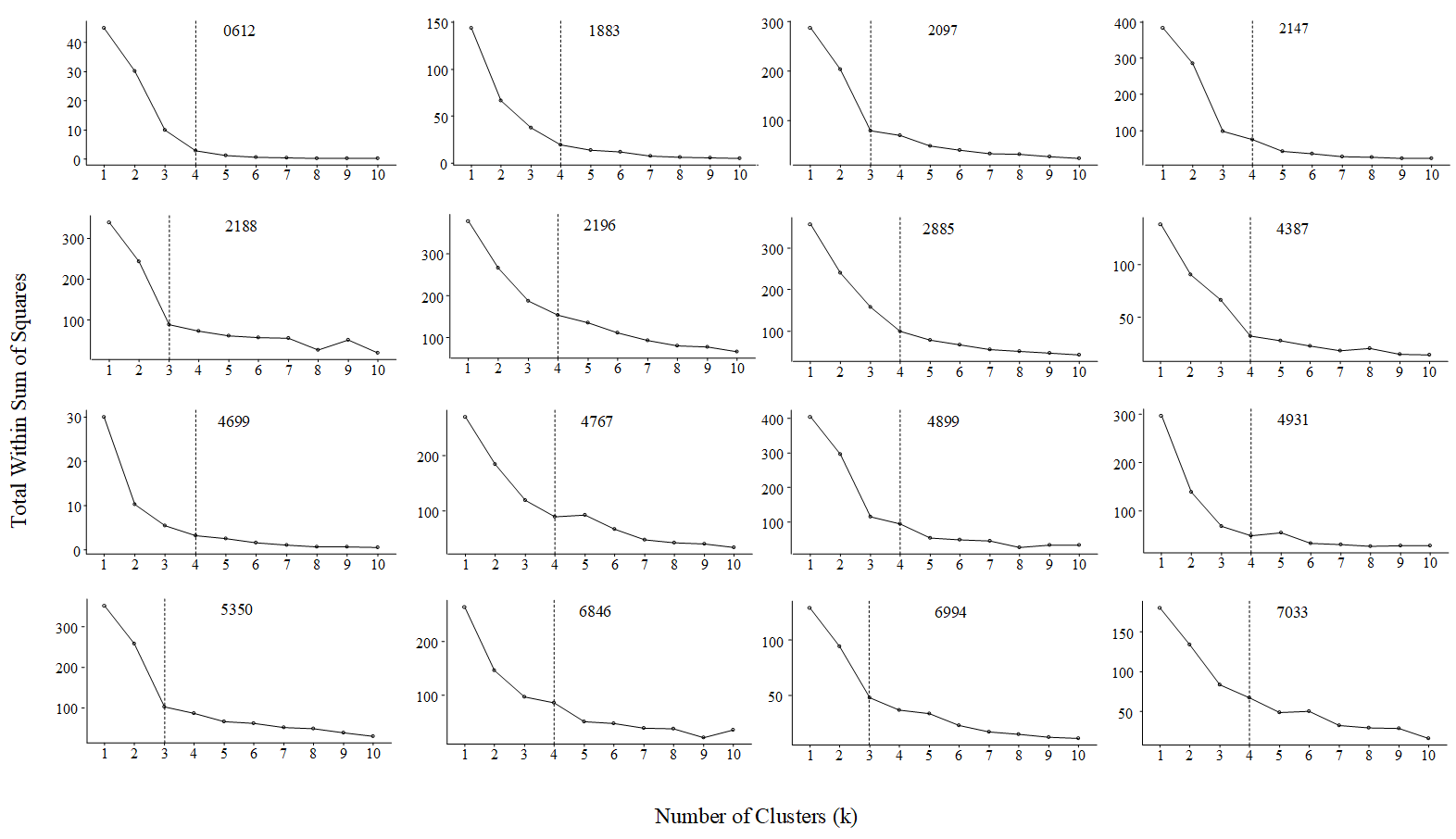
**

**
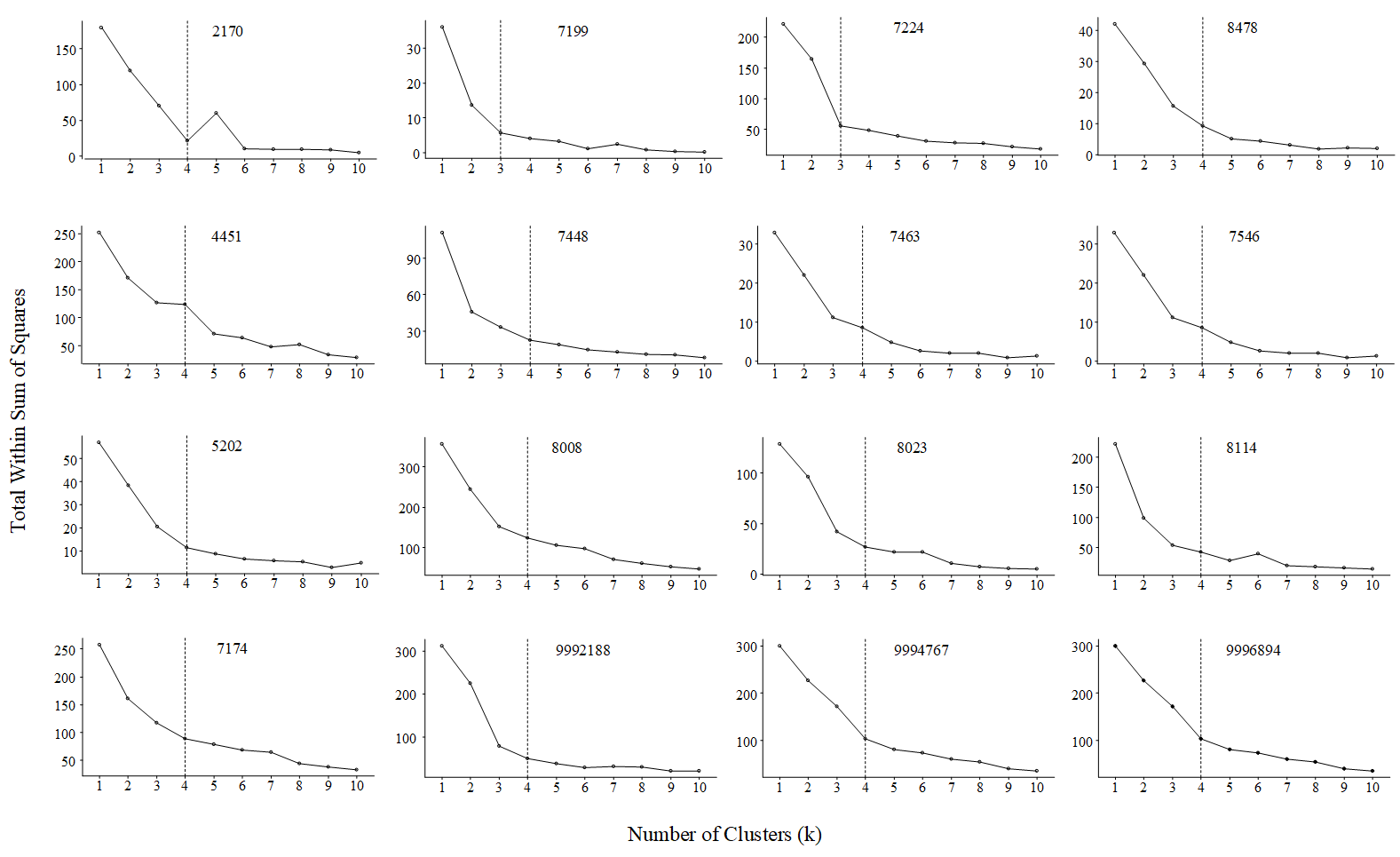
**

**SI Figure 3.**  Proportion, plotted on a log scale, of data points by month in each of four behavioral states identified via telemetry of golden eagles in the Sonoran and Mojave Deserts.
